# Urine-derived Renal Epithelial Cells (URECs) as a source of biomaterial from ciliopathy patients for functional studies and diagnostics

**DOI:** 10.1186/2046-2530-4-S1-P51

**Published:** 2015-07-13

**Authors:** H Ajzeberg, G Slaats, M Stokman, I Logister, N Knoers, R Giles

**Affiliations:** 1 Dept. Nephrology and Hypertension, University Medical Center Utrecht, Utrecht, The Netherlands; 2 Dept. Medical Genetics, University Medical Center Utrecht, Utrecht, The Netherlands

## Objective

Many ciliopathies are diagnosed in infants and children. Obtaining blood and/or skin biopsies for diagnostic and research purposes requires a visit to the clinic and a specialized sample collection skill. We sought alternative non-invasive sources of biomaterial from patients.

## Methods

We have generated an approved Standard Operating Procedure to isolate and culture primary renal collecting duct cells from urine (URECs).

## Results

We show that URECs are approximately 30% proximal tubule cells (megalin positive) and 70% collecting duct cells (AQP2 positive) from the patient. The cells grow on coverslips for immunocytochemistry, can be frozen and grow for 15-20 passages without immortalization. We demonstrate that whereas URECs from healthy individuals ciliate well, URECs from ciliopathy patients with nephronophthisis do not. URECs can form 3D spheroids to study patient-specific tubulogenesis defects. siRNA and overexpression "rescue" experiments in URECs make these cells amenable to functional studies complementing diagnostics. As a proof of principle we show how URECs can be used to test the relevance of genetic variants of "uncertain significance" obtained from whole-exome sequencing. Lastly, we have used patient URECs to explore pharmacological intervention on a patient-specific basis.

## Conclusions

This protocol expands the toolkit available to clinical geneticists and researchers alike in a child-friendly manner. Urine culture offers a non-invasive option for genetic and functional testing and does not require the family to go to the clinic for sample donation. We contend that UREC culture will facilitate personalized medicine for the ciliopathy community and beyond.

